# Extracellular Adenosine-Mediated Modulation of Regulatory T Cells

**DOI:** 10.3389/fimmu.2014.00304

**Published:** 2014-07-10

**Authors:** Akio Ohta, Michail Sitkovsky

**Affiliations:** ^1^New England Inflammation and Tissue Protection Institute, Northeastern University, Boston, MA, USA

**Keywords:** adenosine, A2A-adenosine receptor, A2B-adenosine receptor, regulatory T cell, immunosuppression, tumor microenvironment

## Abstract

Extracellular adenosine-dependent suppression and redirection of pro-inflammatory activities are mediated by the signaling through adenosine receptors on the surface of most immune cells. The immunosuppression by endogenously-produced adenosine is pathophysiologically significant since inactivation of A2A/A2B adenosine receptor (A2AR/A2BR) and adenosine-producing ecto-enzymes CD39/CD73 results in the higher intensity of immune response and exaggeration of inflammatory damage. Regulatory T cells (Treg) can generate extracellular adenosine, which is implicated in the immunoregulatory activity of Tregs. Interestingly, adenosine has been shown to increase the numbers of Tregs and further promotes their immunoregulatory activity. A2AR-deficiency in Tregs reduces their immunosuppressive efficacy in vivo. Thus, adenosine is not only directly and instantly inhibiting to the immune response through interaction with A2AR/A2BR on the effector cells, but also adenosine signaling can recruit other immunoregulatory mechanisms, including Tregs. Such interaction between adenosine and Tregs suggests the presence of a positive feedback mechanism, which further promotes negative regulation of immune system through the establishment of immunosuppressive microenvironment.

## Introduction

Although efficient elimination of pathogens is attributable to the positive feedback nature of immune activation, immune cells also have negative feedback mechanisms that would limit the extent of expansion and effector functions of immune cells. The downregulation of immune response could be not only a homeostatic mechanism, but also an important reaction in protecting vital tissues from non-specific inflammatory damage. Therefore, when the pathogens are cleared, the positive feedback loop of the immune system needs to be broken to save healthy tissues from unnecessary collateral damage. Such endogenous mechanisms terminating inflammation have been a target of research and drug development to modulate the intensity of inflammation.

**Figure 1:**
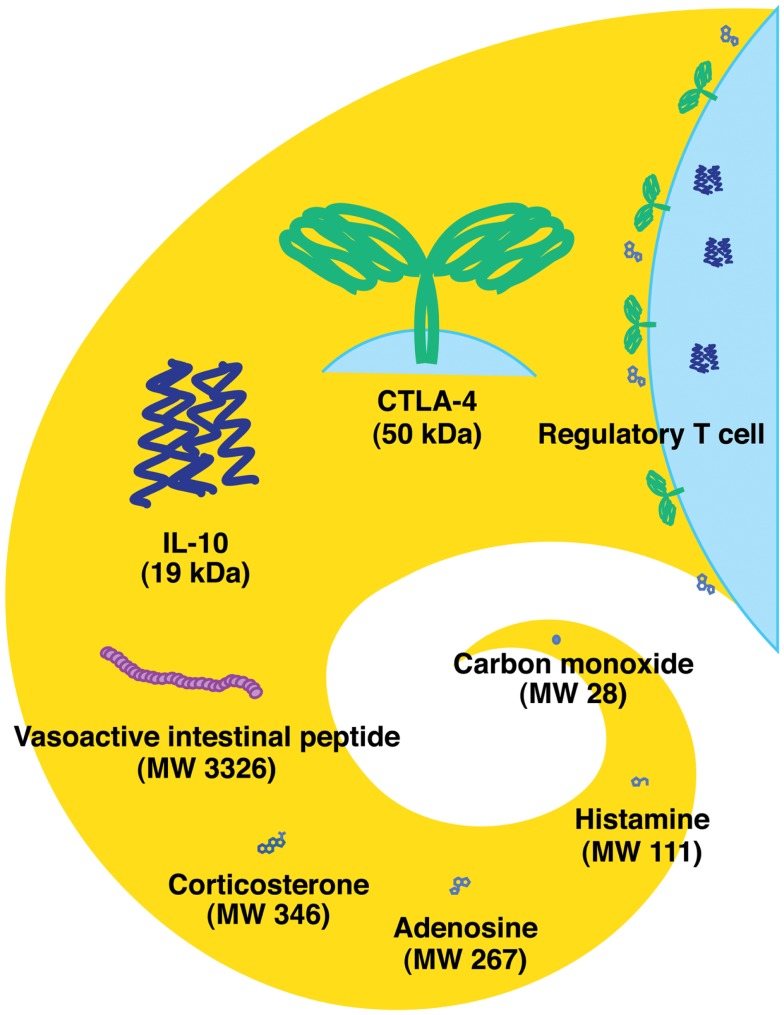
**Endogenous immunoregulatory mechanisms from tiny molecule to cells**. Molecular weight of CTLA-4 represents the approximate size of a homodimer. Tregs express CTLA-4 and produce IL-10 and adenosine.

There are different classes of endogenous anti-inflammatory mechanisms ranging from molecules as small as carbon monoxide to professional suppressor cells including regulatory T cells (Treg) (Figure 1). Small molecules such as prostanoids and glucocorticoids are well-known negative regulators of immune response and are clinically important due to their pharmacological properties. Negative regulators such as anti-inflammatory cytokines (IL-10 and TGF-beta) and cellular proteins [indoleamine-2,3-dioxygenase (IDO), CTLA-4, and PD-1] represent the focus of extensive studies for the last several decades (1, 2). Indeed, blockade of CTLA-4 and PD-1 has currently progressed into promising cancer treatments (3, 4). Some negative regulators of immune response may be produced in response to stress. Extracellular adenosine represents a physiological negative regulator, which increases as a result of metabolic change during hypoxic stress. The intensive interest to adenosine-dependent immunoregulation developed relatively recently.

KEY CONCEPT 1. Extracellular adenosine In the intracellular compartment, adenosine represents an important component of energy metabolism and nucleic acid synthesis. However, extracellular adenosine plays a distinct role in the intercellular signaling via cell surface adenosine receptors.

## Immunoregulation by Endogenous Adenosine

The importance of adenosine receptor signaling has been recognized in the central nervous and cardiovascular systems (5, 6). Pharmacological studies of adenosine receptor signaling in the immune system have established that this pathway has immunosuppressive effects. In general, administration of adenosine or its analogs has been shown to block inflammation in various organs such as the liver, lung, kidney, heart, and digestive tract (7, 8). A variety of inflammatory responses are susceptible to adenosine receptor agonists, especially those capable of stimulating A2A-adenosine receptor (A2AR) (7, 9).

A2AR is ubiquitously expressed in a wide variety of immune cells including T cells, B cells, NK cells, NKT cells, macrophages, dendritic cells, and granulocytes (Table 1). The strong anti-inflammatory function of A2AR implied a possible involvement of its natural ligand, endogenously formed adenosine, in the spontaneous control of immune response. This concept was conclusively proven using A2AR-deficient mice in which the induction of acute hepatitis inflicted much more severe inflammatory tissue damage than in wild-type controls (10). Importantly, the study showed that the lack of A2AR, despite other functional anti-inflammatory mechanisms, was sufficient to exaggerate inflammation, indicating the non-redundant significance of extracellular adenosine in the self-control of inflammatory activities. Exacerbation of various types of inflammation in A2AR-deficient mice generalized the A2AR-dependent control of inflammation (11–14).

**Table 1:** **Adenosine receptor expression in immune cells and signaling pathway**.

	A1	A2A	A2B	A3
Distribution	Dendritic cells Macrophages Neutrophils	Dendritic cells Macrophages Neutrophils Mast cells T cells NK cells NKT cells	Dendritic cells Macrophages Mast cells	Dendritic cells Macrophages Neutrophils Mast cells
Signal transduction	Gi cAMP ↓ PLC ↑	Gs cAMP ↑	Gs/Gq cAMP ↑ PLC ↑	Gi cAMP ↓ PLC ↑

Metabolic changes during inflammation favor the increase of extracellular adenosine (discussed in the next chapter). Inflammation destroys pathogens along with damage to surrounding tissue. In response, adenosine produced from the damaged tissue can suppress proinflammatory activities and prevent further damage. In inflamed and severely hypoxic tissues, local adenosine levels can reach high enough to activate not only A2AR but also low-affinity receptors such as A2B-adenosine receptor (A2BR) (15–17). In many instances, the effect of A2BR stimulation is also immunosuppressive as shown by the inhibition of inflammatory tissue injury by A2BR agonist and by exaggerated inflammation in A2BR-deficient mice (16, 17). A2BR plays a distinctive role in controlling inflammation, e.g., induction of tolerogenic antigen-presenting cells (APC) by alternative activation (18, 19). Thus, the inflammation-related increase of extracellular adenosine initiates negative feedback responses via A2AR and A2BR. The adenosine-A2AR/A2BR pathway serves as an indispensable immunoregulatory mechanism that regulates the extent of immune response.

KEY CONCEPT 2. Alternative activation Alternatively activated macrophages are those activated in a Th2-type cytokine milieu. However, this term is more widely used to represent anti-inflammatory macrophages including those induced in the presence of IL-10, TGF-β or glucocorticoids. Alternatively activated macrophages are involved in resolution of inflammation and tissue remodeling.

## Where Extracellular Adenosine Comes From

**Figure 2:**
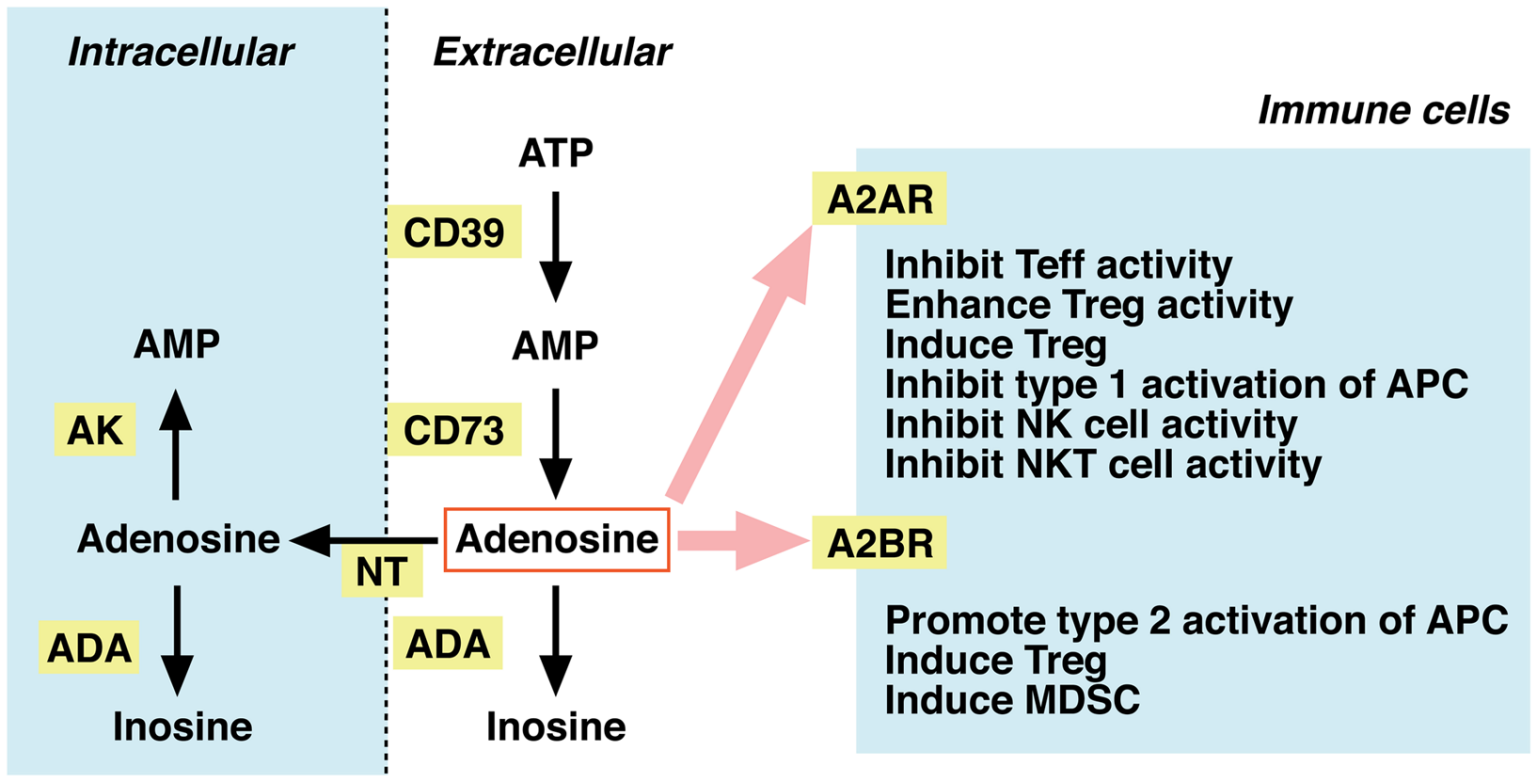
**Metabolism of extracellular adenosine and its effect on cellular immunity**. The activities of CD39 and CD73 produce extracellular adenosine. Extracellular adenosine decreases by adenosine deaminase (ADA)-dependent catabolism and by cellular uptake through nucleoside transporters (NT). Adenosine in the intracellular compartment is converted to AMP by adenosine kinase (AK) or catabolized by ADA. When extracellular levels of adenosine increase, it stimulates A2AR (high-affinity) and A2BR (low-affinity) on immune cells. Adenosine is suppressive to effector T (Teff), NK, and NKT cells. The immunosuppressive activity may be further enhanced by adenosine-mediated induction of Tregs, tolerogenic antigen-presenting cells (APC), and myeloid-derived suppressor cells (MDSC).

Dephosphorylation of ATP results in adenosine formation. In the extracellular compartment, this metabolism is mediated by ecto-5′ -nucleotidases, i.e., CD39 and CD73. CD39 catalyzes degradation of ATP to AMP, and CD73 further converts AMP to adenosine (Figure 2). Extracellular adenosine may be taken up to the intracellular compartment through nucleoside transporters on the plasma membrane, or it may be metabolized to inosine by adenosine deaminase (ADA). Intracellular adenosine may be re-phosphorylated to AMP by the activity of adenosine kinase. Extracellular adenosine concentration is largely reduced in mice lacking CD73, suggesting that degradation of adenine nucleotides is responsible for the production of extracellular adenosine (20, 21). In contrast, inhibitors of ADA and nucleoside transporters increased extracellular adenosine, suggesting the importance of these pathways in the removal of extracellular adenosine (22, 23).

Extracellular adenosine levels are known to increase in the event of inflammation (11, 24–26). This increase may be associated with the release of adenosine and adenine nucleotides in inflamed tissues. Activated polymorphonuclear cells (PMN) were shown to release AMP, which contributed to adenosine increase after metabolism by CD73 (27). It is also likely that inflammatory tissue damage causes uncontrolled leakage of adenine nucleotides from critically damaged cells. Skin irritant was shown to induce ATP and ADP release from keratinocytes (28).

Inflammatory tissue damage, especially damage on vasculature, disturbs blood flow resulting in a diminished oxygen supply. In addition, a massive accumulation of inflammatory effector cells increases local oxygen demand. The deficit in oxygen supply and the increase in oxygen demand cause local hypoxia in inflamed tissue. Tissue hypoxia seems to be conductive to the increase of extracellular adenosine concentration. Hypoxia is known to induce CD39 and CD73 (29, 30) but to inhibit adenosine kinase (31, 32). The increase of adenosine formation and the decrease of removal thus favor adenosine accumulation under hypoxia. Adenosine can also positively regulate CD73 expression and further enhance adenosine formation (33).

Since formation of extracellular adenosine is crucial to downregulation of inflammatory responses, deficiency in adenosine metabolism should affect the intensity of inflammation. Exaggerated inflammation in CD39-deficient and CD73-deficient mice suggested that degradation of extracellular adenine nucleotides by CD39 and CD73 is a major source of adenosine for limiting inflammation (34–37). Similarly, further metabolism of adenosine plays a significant role in controlling the extracellular concentration of adenosine. Inhibitors of ADA and adenosine kinase promote adenosine increase and consequently suppress inflammation (22, 23, 38). The anti-inflammatory effect was also evident after the inhibition of cellular adenosine uptake by nucleoside transporters (39).

## Adenosine Production as a Mechanism of Immunoregulation by Regulatory T Cells

Regulatory T cells are a subset of CD4^+^ T cells expressing CD25 and FoxP3, a transcriptional factor, which regulates the immunosuppressive activity of Tregs. Immunoregulation offered by Tregs is critically important because the lack of Treg leads to the pathogenesis of autoimmune disorders (40, 41). Tregs have various immunosuppressive molecules including TGF-β, IL-10, CTLA-4, and galectin-1, although it is still unclear, which mechanism is the most important for the immunoregulatory activity. Among these mechanisms, Tregs were found to actively produce extracellular adenosine and block activation of effector cells through A2AR (42, 43).

KEY CONCEPT 3. FoxP3 Forkhead box P3 (FoxP3) is a transcription factor that is crucial for development and immunoregulatory function of Tregs. FoxP3 expression is often regarded as a signature of Tregs, especially in mice.

Unlike conventional resting T cells, Tregs were found to express both CD39 and CD73 at high levels (44–46). These nucleotidases on the surface of Tregs were enzymatically active, therefore, Tregs were capable of producing extracellular adenosine from ATP. Since inhibitors of CD39 and CD73 reduced the immunoregulatory activity of Tregs (44, 47, 48), production of adenosine was suggested to represent, at least in part, the immunosuppressive mechanism of Tregs. Tregs were less efficient against A2AR-deficient effector T cells or in the presence of A2AR antagonist (45, 47). These results indicate that adenosine produced from Tregs executes immunosuppression by triggering A2AR-dependent inhibition of effector cell activation. This mechanism is functional in both mice and human Tregs. Human T cells from older people tend to produce larger amount of extracellular adenosine compared to those obtained from younger subjects (49). It might be interesting to study adenosine-dependent immunoregulation by Tregs from the point of view of immunosenescence.

Upon interaction with A2AR, adenosine increases cAMP levels, and subsequent activation of protein kinase A is responsible for the inhibition of cell activation. When Tregs suppress immune response, an increase of cAMP is observed in the target cells (50, 51). Other mechanisms triggering cAMP increase may be also involved in the immunoregulatory activity of Tregs. Indeed, Tregs express cyclooxygenase-2 (COX-2) and produce PGE_2_, which stimulates cAMP production in target cells (52, 53). Along with adenosine, PGE_2_ from Tregs was found to play a role in the immunoregulatory activity of Tregs.

Biological significance of adenosine-dependent immunoregulation by Tregs was demonstrated in various *in vivo* models of inflammatory disorders. While adoptive transfer of wild-type Tregs strongly attenuates inflammation, the transfer of CD73-deficient Tregs could not prevent inflammation including gastritis, acute lung inflammation, ischemia–reperfusion injury, and graft-versus-host disease (14, 48, 54, 55). Similarly, Tregs lacking CD39 failed to block T cell infiltration in contact hypersensitivity (56). In humans, Tregs from AIDS patients and cancer patients express CD39 and CD73 at higher levels than healthy subjects, suggesting adenosine production from Tregs during immunosuppression in humans (57). This evidence increasingly emphasizes the significance of adenosine-producing activity among a variety of immunoregulatory mechanisms of Tregs.

## Regulation of Treg Activity by Adenosine

When the first paper reported adenosine production from Treg, the other group reported an *in vivo* study, which might implicate roles of A2AR in Treg functions (58). In that study, colitis induction by CD4^+^ CD45RB^hi^ naïve T cells and the preventive effect of CD4^+^ CD45RB^low^ cells were examined using T cells derived from wild-type and A2AR-deficient mice. Co-transfer of CD4^+^ CD45RB^low^ cells, which contain Tregs, blocked CD4^+^ CD45RB^hi^ cell-induced pathogenesis; however, colitis by A2AR-deficient CD4^+^ CD45RB^hi^ cells was resistant to the preventive effect of CD4^+^ CD45RB^low^ cell co-transfer. Having been published before the identification of adenosine-producing activity in Tregs, the data might have been enigmatic at that time. Retrospectively, this report might imply immunoregulatory activity of adenosine produced by Tregs presented in CD4^+^ CD45RB^low^ fraction. However, the paper presented other puzzling data indicating that A2AR-deficient CD4^+^ CD45RB^low^ cells were not as effective as wild-type CD4^+^ CD45RB^low^ cells in preventing colitis. Was this data implying that A2AR expression was essential to full activation of Tregs?

It was hypothesized that activity of Tregs might be under control of tissue oxygen tension and extracellular adenosine levels (59). Based on the presence of consensus sequences of hypoxia-responsive element and cAMP-responsive element in the promoter region of anti-inflammatory molecule genes, it was speculated that hypoxia and adenosine would be responsible for the regulation of immunosuppressive activity of Tregs. Although the effect of hypoxia on Tregs is still arguable (60–63), the speculation was proven to be true at least for the adenosine part. When T cells were stimulated with allogenic cells (mixed lymphocyte culture), A2AR agonist strongly inhibited activation of cytotoxic effector T cells. However, in the same cell culture, A2AR agonist massively increased the CD4^+^ FoxP3^+^ population (64). Supporting the hypothesis above, those Tregs expanded in the presence of A2AR stimulation demonstrated an increase in CTLA-4 expression and a significantly stronger immunoregulatory activity (64). Consistent with this observation, pretreatment of Tregs with A2AR agonist before cell transfer enhanced their efficacy *in vivo* in the prevention of ischemia–reperfusion injury. Moreover, A2AR-deficient Tregs were found to be less efficacious in protecting tissues from inflammatory damage, suggesting that endogenous adenosine positively controls the immunoregulatory activity of Tregs *in vivo* (48). A2AR-dependent expansion of Tregs may be important in suppressing inflammatory disorders such as graft-versus-host disease and experimental autoimmune uveitis because the induction of immunoregulatory activity required A2AR expression (65, 66).

KEY CONCEPT 4. Effect of hypoxia on Tregs Hypoxia has been reported to induce FoxP3 in T cells and increase Treg abundance. Such changes are mediated by HIF-1α. However, other reports demonstrated downregulation of Tregs in the presence of HIF-1α, suggesting a complicated role of HIF-1α for Tregs.

The enhancement of immunoregulatory activity may be due to cAMP increase by A2AR stimulation. HIVgp120 binds to CD4 on human Tregs and stimulates immunoregulatory function. This reaction is mediated by the increase of cAMP in Tregs (67). Stimulation of β_2_-adrenergic receptor, which also induces cAMP, can enhance Treg activity as it was observed with A2AR stimulation (68). Conversely, immunoregulatory activity of Tregs attenuates after treatments reducing cAMP levels, e.g., adenylate cyclase inhibition or activation of cAMP phosphodiesterase (69–71). Mechanisms for adenosine-mediated promotion of Treg activity may include recruitment of other anti-inflammatory mechanisms. For instance, COX-2 is inducible by A2AR stimulation to produce potentially anti-inflammatory metabolite PGE_2_ (72). Although A2AR-dependent induction is not directly demonstrated in Tregs, COX-2 is one of the immunosuppressive mechanisms of Tregs as discussed in the previous chapter.

Tregs develop in the thymus (natural Treg) or in the periphery by inducing functional differentiation from conventional T cells (inducible Treg) (40, 41). A2AR stimulation enhanced not only proliferation of natural Tregs but also induction of new Tregs from FoxP3^−^ T cells (64). T cell stimulation in the presence of A2AR agonist induced FoxP3 and LAG3 mRNA in T cells, suggesting newly induced Tregs (73). A2AR agonist further enhanced development of inducible Tregs by TGF-β (65). In addition to A2AR, A2BR also plays a role in the induction of Tregs. Agonist of A2BR promoted, but A2BR-deficiency prevented, Treg induction (74). In human Tregs, vasoactive intestinal peptide (VIP) was shown to promote Treg induction via cAMP (75). It is possible that the adenosine-dependent induction of Tregs may be again mediated by cAMP induction.

**Figure 3:**
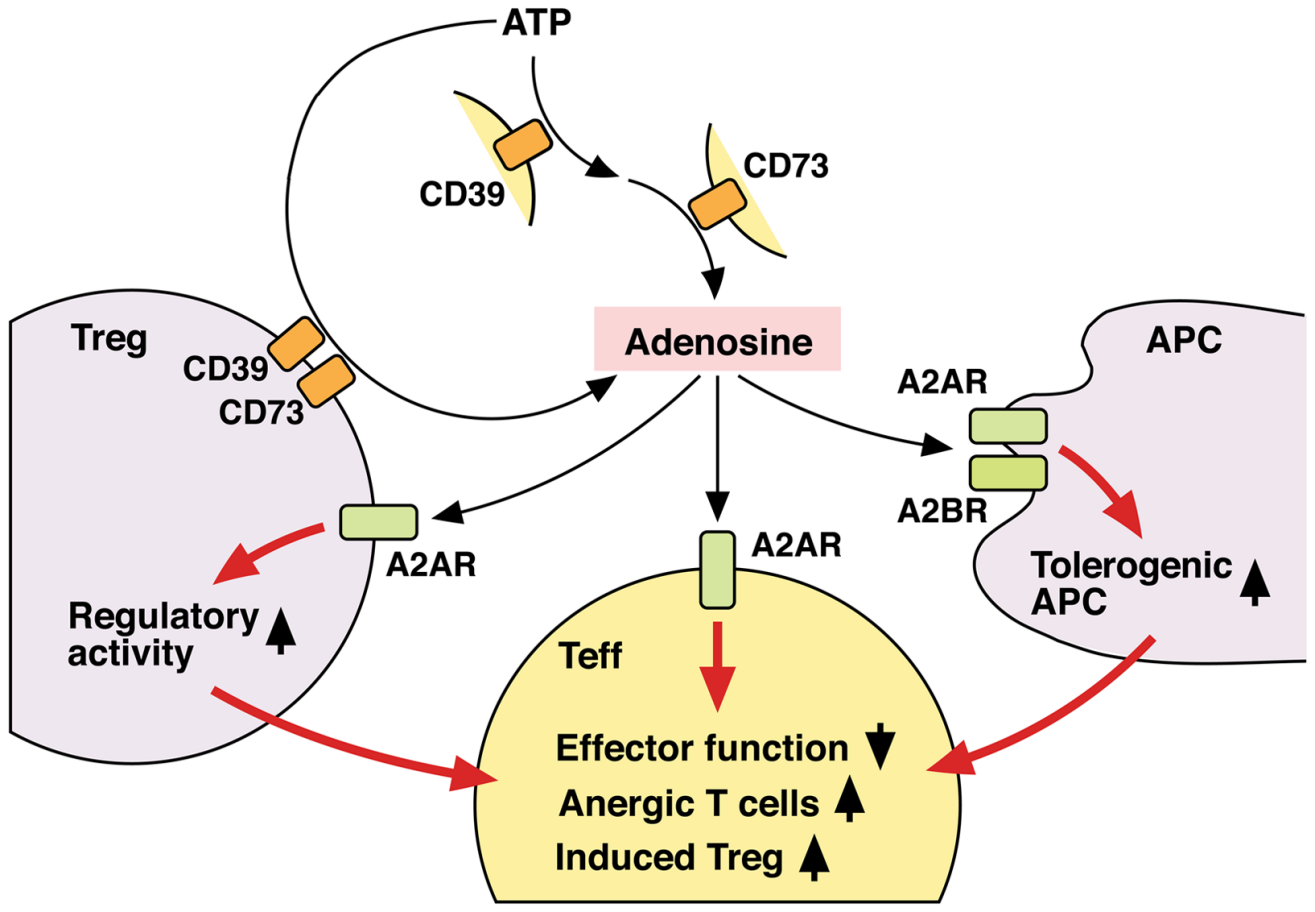
**Mechanisms of T cell regulation by extracellular adenosine**. Extracellular adenosine can be produced by activities of CD39 and CD73 on cell surface. And its interaction with A2AR directly inhibits T cell activation. Tregs express both CD39 and CD73 at high levels and use adenosine for their immunoregulatory activity. Adenosine enhances immunoregulatory activity of Tregs via A2AR signaling. A2AR signaling in effector T cells may induce differentiation into Tregs. There might be a positive feedback loop between adenosine and Treg-dependent immunoregulation. Moreover, adenosine increases tolerogenic APCs, which are poor stimulators of effector T cells. Thus, adenosine suppresses T cell immunity both by directly inhibiting activation of effector T cells and indirectly by producing the immunosuppressive environment. By employing different mechanisms, the immunosuppression by adenosine might be quickly effective and persistent.

Thus, Tregs not only utilize adenosine as one of their immunosuppressive mechanisms, but also receive positive regulation from adenosine to enhance the number and immunosuppressive activity of Tregs (Figure 3). Since both Tregs and adenosine modify immune response in the negative direction, it seems reasonable that these two elements are mutually enhancing their production and activity.

## Modulation of Treg Activity through the Intervention to Adenosine-A2AR Pathway

Excess adenosine in ADA deficiency causes a detrimental effect on immune cells and results in severe combined immunodeficiency. Pegylated ADA (PEG-ADA) has been used for treatment by decreasing adenosine levels in these patients. A recent report suggested a decrease of Treg activity in mice and humans after treatment with PEG-ADA (76). It may be possible to manipulate Treg activity *in vivo* through intervention in the adenosine-A2AR pathway.

Conversely, adenosine-producing CD73 is inducible by TGF-β (77). TGF-β induces Tregs, and the increase of adenosine-producing activity may contribute to the enhancement of immunoregulatory activity of the induced Tregs. In addition to the induction of Tregs, this cytokine is also involved in functional differentiation of naïve CD4^+^ T cells into Th17 cells. Although Th17 cells have been known for their proinflammatory activities, they indeed express CD39 and CD73 and are capable of suppressing T cell activation by producing adenosine (78). Interestingly, such function was found only in Th17 cells induced by TGF-β + IL-6, whereas those induced in the absence of TGF-β were not immunosuppressive. CD73-inducing activity of TGF-β may be responsible for this difference. In addition to TGF-β, various agents including triiodothyronine (T3), IFN-α, indomethacin, and rosuvastatin are capable of inducing CD73 (79–82). Interestingly, anti-inflammatory action of some clinical medications is explained by the increase of adenosine. Methotrexate (83, 84) and sulfasalazine (85, 86) can increase adenosine concentration high enough to suppress inflammatory response through A2AR. This increase in adenosine is dependent on CD73 activity. It is possible that clinical use of such agents promote immunoregulation by Tregs. Indeed, among these CD73 inducers, statins were shown to increase the number and function of Tregs (87, 88).

The promotion of Treg-dependent immunoregulation should be beneficial to alleviate many inflammatory disorders and to facilitate successful tissue transplantation (89, 90). In hematopoietic stem cell transplantation, transfer of Tregs should be able to suppress graft-versus-host disease, which is caused by the attack of recipient-derived lymphocytes to the host cells and is occasionally lethal (90, 91). Adenosine receptor stimulation will be useful to increase the recovery of Tregs during *in vitro* expansion and to promote their efficacy after the transfer.

While immunoregulation by Tregs is crucial to prevent autoimmunity, ironically, the same mechanism benefits tumor tissue by providing protection against immune attack. Accumulation of Tregs represents the immunosuppressive nature of the tumor microenvironment, and elimination of Tregs improves immunological tumor regression (1, 2, 92). Adenosine was also demonstrated to accumulate in tumors (93, 94). Tissue hypoxia, which is conductive to the increase of extracellular adenosine levels, is not uncommon in tumors due to the disorganized proliferation of tumor cells and poor blood flow (95, 96). The significance of adenosine in tumors was demonstrated when A2AR-deficient mice, but not wild-type mice, underwent complete regression of solid tumor (94). The tumor-protective role of adenosine was further demonstrated by retarded growth or enhanced elimination of tumors in A2BR-deficient mice (97, 98) and CD73-deficient mice (99, 100). Adenosine can directly down-regulate effector functions of anti-tumor immune cells and may also indirectly suppress anti-tumor immune response by promoting Treg activities. Moreover, among other effects of adenosine is the preferential alternative activation of APCs (18, 19) and induction of myeloid-derived suppressor cells (101), both of which lead to inactivation of immune cells. Wrapped in extracellular adenosine, tumor cells employ multiple mechanisms to evade anti-tumor immune response.

KEY CONCEPT 5. Tumor microenvironment It has long been a question why immunotherapy mightily struggles against tumors *in vivo* even with highly active immune cells. Tumors contain a number of immunosuppressive mechanisms and inactivate incoming anti-tumor immune cells. Countermeasure to the immunosuppression in tumor microenvironment is complementary to the current protocol of immunotherapy and is expected to improve tumor regression.

KEY CONCEPT 6. Myeloid-derived suppressor cells A population of myeloid-derived suppressor cells (MDSCs) includes immature forms of macrophages, granulocytes and dendritic cells. MDSCs express immunosuppressive molecules (arginase and reactive oxygen/nitrogen species) and strongly suppress T-cell activities.

Maintaining the capability of eliminating cancer cells by host immune cells is a great advantage in the effective treatment of cancer. Anti-tumor immunity is expected to have a positive impact either alone or complementary to surgical removal of the tumor mass because immune cells should be able to seek out and destroy hidden and metastatic cancer cells. Improving anti-tumor T cell activity by A2AR antagonists (94, 102, 103) and CD73 inhibitors (102, 104) suggests promise for disengaging the adenosine-mediated immunosuppression in the tumor microenvironment.

**Figure 4:**
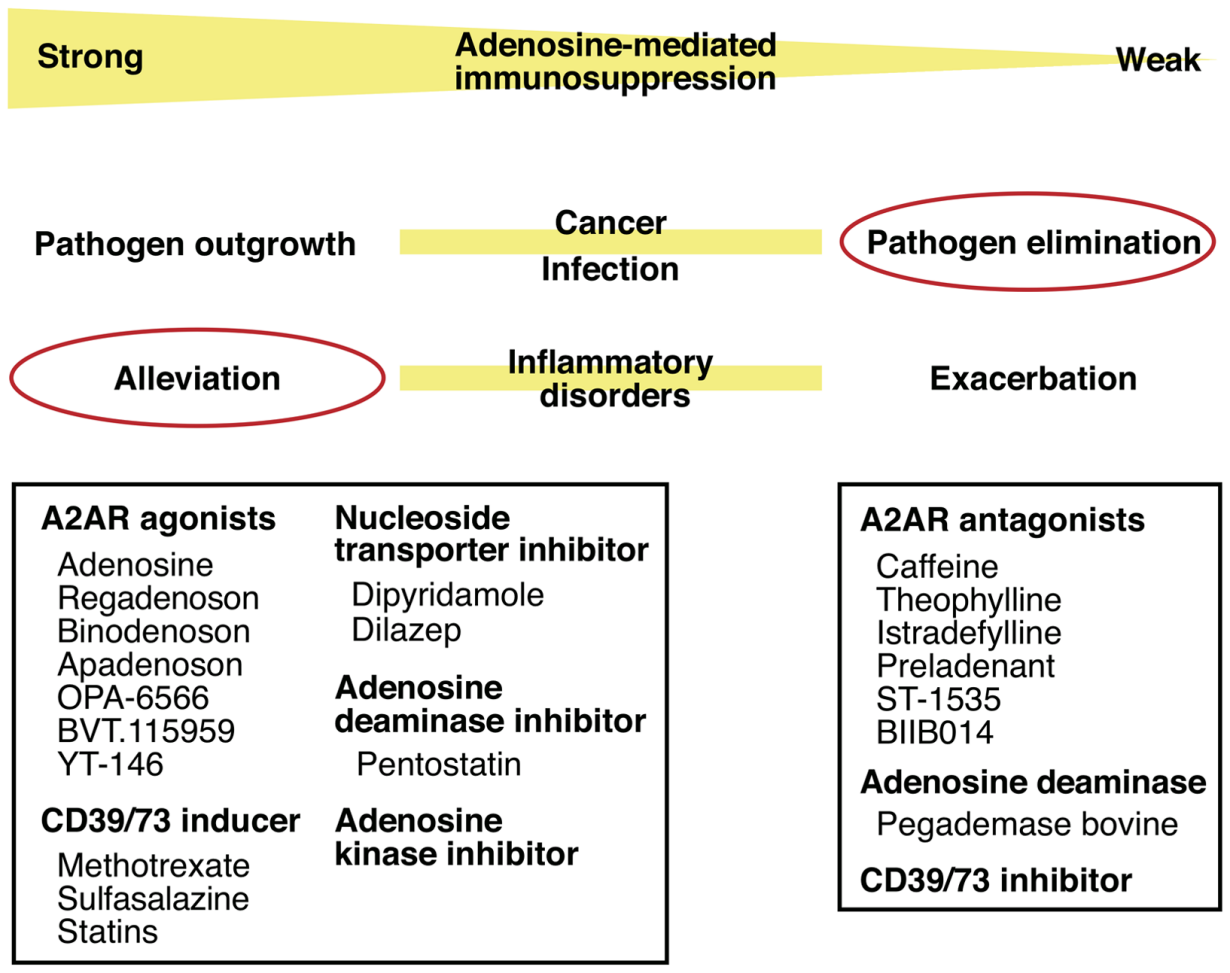
**Intervention in pathophysiological conditions by modulating the adenosine-A2AR pathway**. Enhancement of the adenosine-mediated immunosuppression can be done by directly stimulating A2AR using agonists or by increasing adenosine levels using CD39/73 inducer or inhibitors of nucleoside transporter, adenosine deaminase, and adenosine kinase. This treatment should alleviate various inflammatory disorders. Conversely, weakened adenosine-dependent immunosuppression is expected to enhance anti-pathogen immune response. This modulation will be possible by inhibiting extracellular adenosine production (CD39/73 inhibitor), by enhancing adenosine degradation (adenosine deaminase), or by blocking adenosine binding to A2AR (antagonists).

Thus, intensity of inflammation may be manipulated by intervening in the adenosine-A2AR/A2BR pathway. The direction of manipulation, either inhibition or promotion, will be dependent on the nature of disease. Enhancement of the adenosine-mediated immunoregulation will be beneficial to treat inflammatory disorders such as acute lung injury, arthritis, inflammatory bowel diseases, and diseases accompanying ischemia–reperfusion injury. The same strategy may promote successful hematopoietic stem cell transplantation by blocking graft-versus-host reaction and tissue transplantation by inhibiting ischemia–reperfusion injury and allograft rejection. The inhibition of this mechanism will promote elimination of pathogen (Figure 4).

## Mechanisms of T Cell Inhibition by Adenosine

The predominance of A2AR on T cells has been known for years (105), but it took some time before T cells attracted more attention as the important target of A2AR-mediated immunosuppression. A2AR agonists block T cell activation by interfering with T cell receptor signaling (106, 107), and they inhibit proliferation and effector functions of T cells such as cytotoxicity and cytokine-producing activity (9, 108, 109). Experiments with purified CD4^+^ and CD8^+^ T cells showed that stimulation of A2AR on T cells could directly suppress their activation (109). This direct action of adenosine can instantly suppress inflammatory responses in extensively damaged tissue to save the tissue from critical loss of function. Recent studies suggest that adenosine regulates T cell activities in different levels (Figure 3).

T cell activation in an adenosine-rich environment allows expansion of activated T cells lacking effector functions. Indeed, T cells activated in the presence of A2AR agonist could produce a small amount of IFN-γ even after the removal of A2AR agonist (73, 109). The impairment of effector function was persistent in these T cells. Therefore, immunoregulation by adenosine is not simply an immunosuppressive effect in its very presence, but it imprints T cells with longer-lasting memory to the immunosuppressive signal, i.e., anergic T cells.

In addition, adenosine can induce APCs that are capable of producing immunosuppressive molecules such as TGF-β, IL-10, arginase, IDO, and COX-2 (18, 19). Induction of these molecules indicates alternative activation of APCs, which leads to the inhibition of T cell activation. While A2AR mediates inhibition of classical proinflammatory activation of APCs, A2BR may play a major role in the induction of alternative activation. Indeed, APCs stimulated in the presence of adenosine became a tolerogenic phenotype that is quite inefficient in producing effector T cells. Furthermore, adenosine may suppress antigen-specific activation of T cells by interfering with the migration of T cell and APCs in the draining lymph node (110).

As we discussed, adenosine promotes expansion of Tregs and their immunoregulatory activity. The rise of professional immunoregulatory cells would be of great importance in the adenosine-inducible immunosuppressive environment. Immune activation in the presence of adenosine can establish a memory of exposure to the immunosuppressive signal.

Most of these cellular reactions to adenosine seem to be mediated by cAMP. Both A2AR and A2BR are coupled to Gs protein, and stimulation of these receptors can increase cAMP production by adenylate cyclase (Table 1). The increase of cAMP activates protein kinase A, but activation of Epac also happens at least after A2AR stimulation (111). The adenosine receptor signaling pathway that results in promotion of Treg activity is yet to be elucidated.

Research has revealed that adenosine is capable of regulating a wide range of immunoregulatory mechanisms. Notably, adenosine actively promotes Treg-mediated immunoregulation by increasing cell number and by enhancing their activity. Thus, immunosuppression by adenosine involves quick, counteractive, and direct inhibition of immune activation and long-term effect, e.g., anergic T cells, tolerogenic APCs, Tregs, and myeloid-derived suppressor cells. By evoking all these mechanisms, adenosine may play an important role in establishing an immunosuppressive environment, which can be seen in tumors. It is also interesting that adenosine acts as a regulator of other endogenous immunoregulatory mechanisms.

## Conflict of Interest Statement

The authors declare that the research was conducted in the absence of any commercial or financial relationships that could be construed as a potential conflict of interest.
